# Use of a digital health solution after percutaneous coronary intervention: a randomised controlled trial

**DOI:** 10.3389/fdgth.2026.1851777

**Published:** 2026-07-21

**Authors:** Elias Freyr Gudmundsson, Bartosz Dobies, Brynja Laxdal, Inga Valborg Olafsdottir, Ingibjörg Davidsdottir, Heida Birna Bragadottir, Svala Sigurdardottir, Ari Pall Isberg, Andrew Grannell, Berglind Libungan, Brynjolfur A. Mogensen, Hulda Halldorsdottir, Maria V. Gudmundsdottir, Bylgja Kaernested, Tryggvi Thorgeirsson, Saemundur Jon Oddsson, David O. Arnar

**Affiliations:** 1 Sidekick Health, Reykjavik, Iceland; 2 Doctoral School of Medical and Health Sciences, Jagiellonian University Medical College, Krakow, Poland; 3 Cardiovascular Services, Landspitali University Hospital, Reykjavik, Iceland; 4 School of Health Sciences, University of Iceland, Reykjavik, Iceland; 5 Center for Mindfulness, Clinical Medicine, Aarhus University, Aarhus, Denmark

**Keywords:** cardiac rehabilitation, coronary artery disease, digital health, digital therapeutics, percutaneous coronary intervention, remote patient monitoring, secondary prevention

## Abstract

**Aims:**

To evaluate a multimodal digital health intervention (SK-121) as an adjunct to standard of care (SoC) to reduce the risk of recurrent cardiovascular events in patients following percutaneous coronary intervention (PCI).

**Methods and results:**

In this single-centre, randomised controlled trial of 200 patients (mean age 64.4 years; 79.5% male) who underwent PCI, patients were randomised 1:1 to receive SoC alone or SoC plus a 48-week gamified digital program focusing on lifestyle modification and remote patient monitoring (RPM). User retention and engagement were high with 84% remaining active at 24 weeks and weekly RPM completion averaging 94%. The primary endpoint was the baseline-adjusted between-group difference in the Second Manifestations of ARTerial disease, version 2 (SMART2) risk score at 6 months. These SMART2 scores did not differ between the intervention and control groups (adjusted mean difference −0.3%; 95% confidence interval −2.4% to 1.9%; *P* = 0.821). However, the intervention group significantly improved in disease knowledge at 6 and 12 months (*P* < 0.001) and medication adherence at 6 months (*P*=0.030). Diastolic blood pressure was significantly lower in the intervention group at 12 months (−3.7 mmHg; *P* = 0.003). In a *post hoc* exploratory hypothesis-generating subgroup analysis of overweight participants, the intervention significantly reduced SMART2 scores at 12 months compared to controls (−3.7%; *P* = 0.033).

**Conclusion:**

While the intervention did not reduce the SMART2 risk score in the overall population, it significantly improved disease knowledge, short-term medication adherence, and diastolic blood pressure. The program was well-accepted and may offer prognostic benefits for overweight patients.

**Clinical Trial Registration:**

https://clinicaltrials.gov/study/NCT05713565, NCT05713565.

## Introduction

Coronary artery disease (CAD) remains a major threat to global public health, accounting for approximately 8.9 million deaths annually (1). Up to 90% of CAD and myocardial infarction risk is attributable to modifiable factors, including unhealthy diet, physical inactivity, obesity, and smoking (2–4).

A significant gap persists between clinical guidelines for CAD and real-world implementation (5–7). Therapy for CAD should therefore, in addition to medical intervention, include support for lifestyle modifications. Exercise therapy is, for instance, a cornerstone of cardiac rehabilitation, favourably impacting key CAD determinants, including autonomic tone, blood pressure, and metabolic profiles (8, 9), while potentially contributing to reversal of the atherosclerotic processes (10).

Digital health technologies offer a scalable solution to improve adherence to lifestyle modifications and bridge the gap between acute care and long-term maintenance. The digital therapeutics company Sidekick Health (SKH) developed, in collaboration with cardiovascular experts, a digital health program (SK-121) for patients with CAD to support the adoption of cardiovascular risk-reducing lifestyle behaviours. Concurrent with the standard of care (SoC), patients are supported with personalised, disease-specific education and treatment to adopt healthy lifestyle habits across nutrition, exercise, psychological well-being, and sleep, in addition to remote patient monitoring (RPM). Behavioural change techniques such as goal setting, action plans, and feedback are central to the program, along with self-monitoring of medication use and health-related metrics. Similar digital programs developed by SKH for other diseases were found to be user-friendly, engaging, and associated with improved health outcomes (11–13).

We evaluated in a randomised controlled trial (RCT) whether a holistic approach delivered through a multimodal digital health program adjunct to SoC after a percutaneous coronary intervention (PCI) for CAD would improve the predicted risk of recurrent cardiovascular events.

## Methods

### Study design and ethics

This single-centre randomised controlled trial was conducted at the Cardiology outpatient clinic at Landspitali University Hospital in Reykjavik, where all patients received guideline-recommended SoC therapy. A total of 200 participants were recruited between February 6, 2023, and December 14, 2024, and screened for eligibility by research nurses or physicians. Written informed consent was obtained from all participants before any study-related procedures. Data were collected at baseline, 6 months, and 12 months. Due to the nature of the intervention, blinding of patients, nurses, and physicians was not feasible. However, the statistical analyses were based on a pre-specified statistical analysis plan (SAP) and performed by a statistician who was blinded to the group allocation. The study protocol was approved by the Icelandic National Bioethics Committee (application number: VSN-22-109) and the Scientific Research Committee for Health Research at Landspitali University Hospital. The trial was registered on ClinicalTrials.gov (NCT05713565) on the 6th of February 2023.

### Eligibility criteria

Participants were eligible for inclusion if they were adults (≥18 years) with a diagnosis of CAD, including both incident and recurrent cases of non-ST-segment elevation myocardial infarction (NSTEMI), ST-segment elevation myocardial infarction (STEMI), or acute coronary syndrome (ACS), who had undergone PCI within 7–30 days prior to enrolment. This specific timeframe was selected to align with the participants' first routine outpatient clinic visit; the exact timing of this visit varied based on a number of clinical factors, including the complexity of the PCI procedure, but all patients were routinely scheduled for an outpatient evaluation within 30 days of their intervention.

All participants were pre-screened and registered by cardiologists or nurses based on records from the Cardiology service at Landspitali University Hospital in Iceland. Additional inclusion criteria were to be able to understand written and verbal instructions in Icelandic, to own and operate a smartphone, and to be willing and able to comply with the study protocol and attend scheduled visits. Participants were excluded if they did not own a smartphone compatible with the SKH app, were unwilling to install or use the app, or were unable to operate it. Additional exclusion criteria included the presence of other serious medical conditions (e.g., cancer, endocarditis, heart failure with ejection fraction <40%, history of cardiac arrest) or active alcohol or drug abuse.

### Randomisation

After obtaining informed consent, baseline data were collected by clinical staff during the initial clinic visit. Upon enrolment, participants were randomised in a 1:1 ratio to either the intervention group, which received the SKH app in addition to SoC, or the control group, which received SoC only. For randomisation participants were stratified by sex and troponin T elevation (yes/no) to ensure balance concerning PCI-only events vs. STEMI/NSTEMI. Participants were randomised using electronic Case Report Form (eCRF) software (Greenlight Guru Clinical), and baseline information was entered into the system by the research nurses.

### Intervention

**Figure 1:**
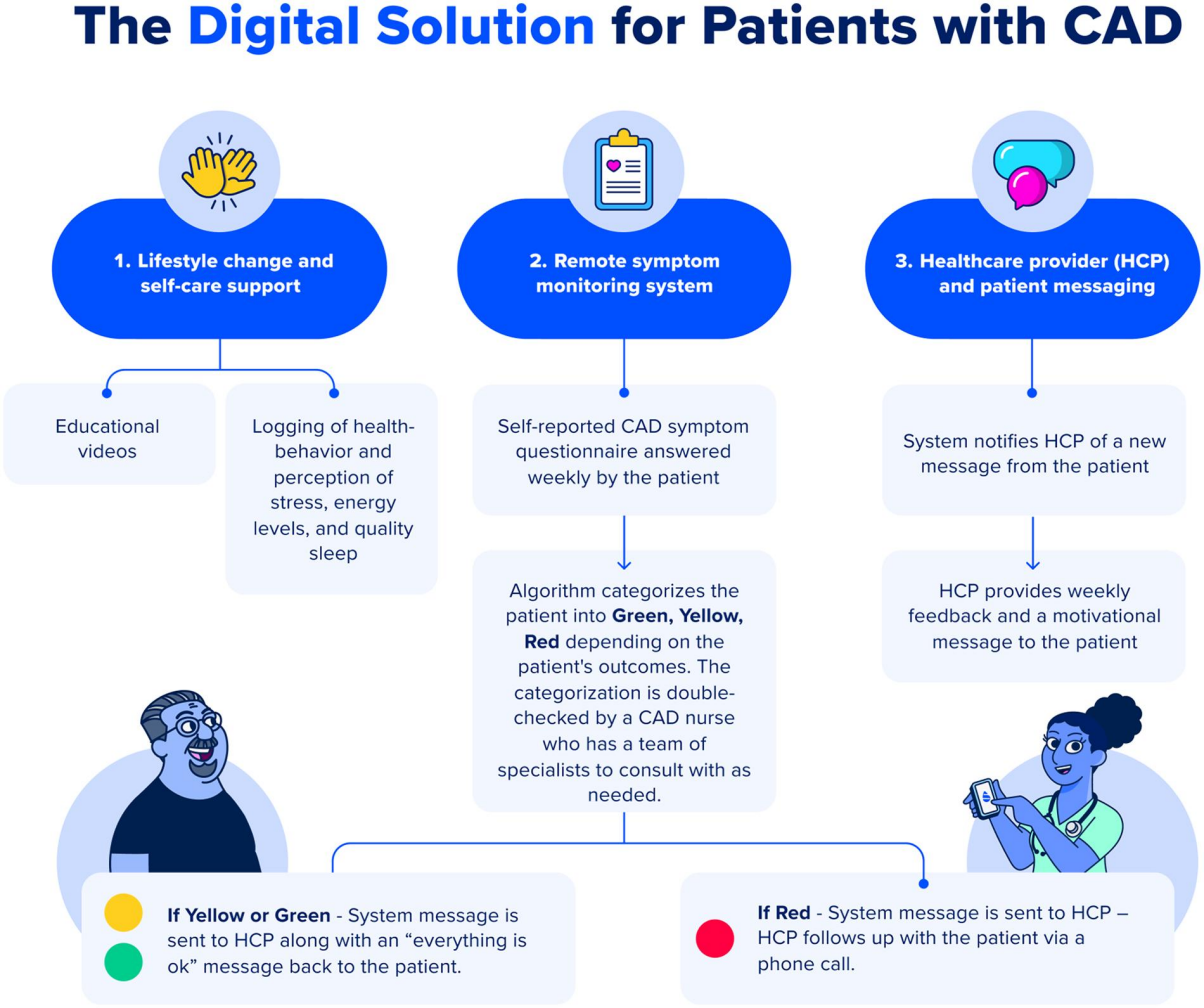
Overview of the SKH digital solution for CAD patients. CAD, coronary artery disease; HCP, healthcare professional.

The digital intervention (Sidekick Health, SK-121) is a 48-week, remotely delivered program (Figure 1) designed to complement post-PCI care. It uses a salutogenic, strengths-based approach to support sustainable health-promoting behaviours. The program features a 24-week active phase followed by a 24-week maintenance phase, grounded in self-determination theory, utilising goal setting, feedback, gamification, and altruistic rewards. The program's gamification was driven by daily “missions,” which prompted participants to complete tasks such as reading educational content cards, watching instructional videos, or tracking cardiovascular symptoms.

During the onboarding phase (weeks 1–4), participants received guidance on app use, and introductory education on CAD and secondary prevention. An optional smoking-cessation pathway was available if relevant. Thereafter, programme content was dynamically tailored to individual needs, assessed at regular intervals through in-app questionnaires. The remaining 20 active weeks provided coaching and modules on nutrition, mental health, sleep, stress management, and exercise. In addition, disease-specific content and material addressing psychosocial aspects of living with CAD were provided, including social connectedness, intimacy, alcohol use, and return-to-work considerations.

Clinical safety was integrated through weekly remote monitoring of angina and dyspnoea. Participants were also encouraged to regularly report symptoms, blood pressure, and peripheral oedema. CAD-nurses reviewed symptom reports, and throughout the programme, a health coach provided weekly written individual feedback on behavioural progress.

During the maintenance phase, participants continued to receive recurrent educational content, motivational prompts, and symptom monitoring to support consolidation of behavioural changes and long-term self-management (Figure 1). Partners or close family members were offered optional access to the programme to enhance understanding of CAD and to support the participant's daily self-management and recovery.

Evaluations included 6-month engagement and weekly RPM questionnaires covering symptoms, side effects, and medication adherence. A tiered algorithm (green/yellow/red) guided clinical responses, with red triggering immediate contact (Figure 1). Engagement metrics included active days, mission completion, and medication reminders, with retention defined as activity at least once per week. Users also provided feedback on app improvements and recommendations.

### Endpoints and measurements

The primary endpoint was the baseline-adjusted between-group difference in the SMART2 risk score [expressed as predicted absolute risk reduction (ARR)], which estimates the 10-year risk of recurrent atherosclerotic cardiovascular disease (ASCVD) and ranges from 0% to 100%. The SMART2 risk score differs from the original SMART score (14) by incorporating updated data from more contemporary populations, adjusting for regional differences in cardiovascular disease (CVD) incidence, and accounting for competing risks of non-CVD death (15).

The SMART2 score was calculated with the formula provided by Hageman et al., using the regional adjustment for Iceland (“Europe, moderate risk region”) (15). Patients were further categorised based on their predicted 10-year SMART2 risk as follows: low (<10%), moderate (10% to <20%), high (20% to <30%), very high (30% to <40%), and extremely high (≥40%) (14, 16).

Secondary endpoints included between-group differences in scores on the following questionnaires: the 20-item short version of the Coronary Artery Disease Education Questionnaire (CADE-Q SV), which assesses cardiovascular patients' knowledge across five domains (diet, exercise, clinical aspects, psychosocial risk, and risk factors) (17); the 8-item Morisky Medication Adherence Scale (MMAS-8) (a license was obtained for this study, see acknowledgements) (18–20); the 21-item Depression, Anxiety, and Stress Scale (DASS-21) (21); and the 7-item Seattle Angina Questionnaire (SAQ-7), which evaluates three domains: physical limitation, angina frequency, and quality of life (22). CAD-related hospital utilisation was compared for the groups 12 months prior to and after enrolment into the study.

The exploratory endpoints pre-specified in the study protocol and SAP included between-group differences in the following outcomes: quality of life assessed using the 5-level EuroQol-5D questionnaire (EQ-5D-5L), rescaled from 0 to 1 to 0–100 range; overall health status assessed with the EuroQoL visual analogue scale (EQ-VAS) (23); weight (in kg); body mass index (BMI, in kg/m^2^); waist circumference (in cm); waist-height ratio; physical function assessed by the number of repetitions in the 30-second sit-to-stand test (24); the number of metabolic syndrome components present, i.e., elevated triglycerides, high fasting glucose, low high-density lipoprotein (HDL) cholesterol, increased waist circumference, and elevated systolic blood pressure (25); lipoprotein(a) (in nmol/L); high-sensitivity C-reactive protein (hs-CRP; in mg/L); estimated glomerular filtration rate (eGFR; in mL/min/1.73 m^2^); HDL cholesterol (in mmol/L); triglyceride-to-HDL ratio; and three liver enzymes (in U/L): alanine transaminase (ALT), aspartate aminotransferase (AST), and gamma-glutamyl transferase (GGT).

### Sample size calculation

Prior research is limited for assessing the marginal risk reduction of cardiovascular events achieved through risk reduction strategies in secondary prevention (26). A study of 281 patients with acute coronary syndrome estimated that achieving guideline-recommended targets for all modifiable risk factors would result in a median SMART risk score reduction from 16.1% to 9.6%, corresponding to an ARR of 6.5% (27). Thresholds for minimal clinically important differences in SMART-based risk scores are lacking, but an ARR of at least 5% can be meaningful in the context of secondary cardiovascular prevention.

We expected enrolled patients to be at elevated cardiovascular risk due to their underlying condition of CAD and having undergone PCI within the last 30 days. Based on this, we anticipated a between-group difference of approximately 5% in the SMART2 risk score (scaled 0–100), with an estimated pooled standard deviation of 12.

Using G*Power for sample size estimation, a total of 180 patients would provide 80% power to detect a difference at a two-sided alpha of 0.05. To account for an anticipated dropout rate of approximately 10%, we recruited an additional 20 participants, bringing the final sample size to 200.

### Statistical analysis

Statistical analyses were performed using R (version 4.4.0), with a significance level of *α* = 0.05 for two-sided tests. Baseline characteristics were summarised using descriptive statistics: continuous variables as means with standard deviations (SD) or medians with interquartile range (IQR), and categorical variables as frequencies and percentages. The internal consistency of multi-item questionnaires (CADE-Q SV, MMAS-8, DASS-21, and SAQ-7) was assessed using Cronbach's alpha. Missing data were handled using multiple imputation by chained equations with 20 imputations and 20 iterations per outcome (*mice* package, version 3.16.0). Predictive mean matching was used as the imputation method, and the imputation model included all variables used in the analysis models. Endpoints were assessed using an intention-to-treat (ITT) approach, including all participants who completed at least one study visit (including baseline).

The primary endpoint (SMART2 score) was analysed using a generalised linear mixed-effects model (*glmmTMB* package, version 1.1.10), with a beta distribution and logit link to account for the bounded, right-skewed, and continuous nature of the outcome.

Secondary and exploratory endpoints were analysed using linear mixed-effects models (*lmerTest* package, version 3.1.3) or robust linear mixed-effects models (*robustlmm* package, version 3.3.1) when Q-Q plots of random effects indicated deviations from normality, such as heavy tails or outliers. Models were adjusted for the baseline value of the respective outcome, age, sex, disease status before enrolment (i.e., stable angina/ambulant, unstable angina, NSTEMI, STEMI), and whether the index PCI was the first CAD diagnosis.

For the primary endpoint, an additional unadjusted model (i.e., adjusted only for the baseline value of the outcome) was also presented. Time-by-group interactions were included in all models, and participant-specific random intercepts were used to account for within-subject correlation over time. Estimated marginal means and between-group contrasts at each time point were derived using the *emmeans* package (version 1.10.3), based on the fitted models and stratified by time to evaluate both 6- and 12-month effects.

All results were presented with 95% confidence intervals (CI). No formal correction for multiple comparisons was applied; therefore, given the exploratory nature of the secondary and exploratory endpoints, the results should be interpreted with caution and as hypothesis-generating.

## Results

### Participants

**Figure 2:**
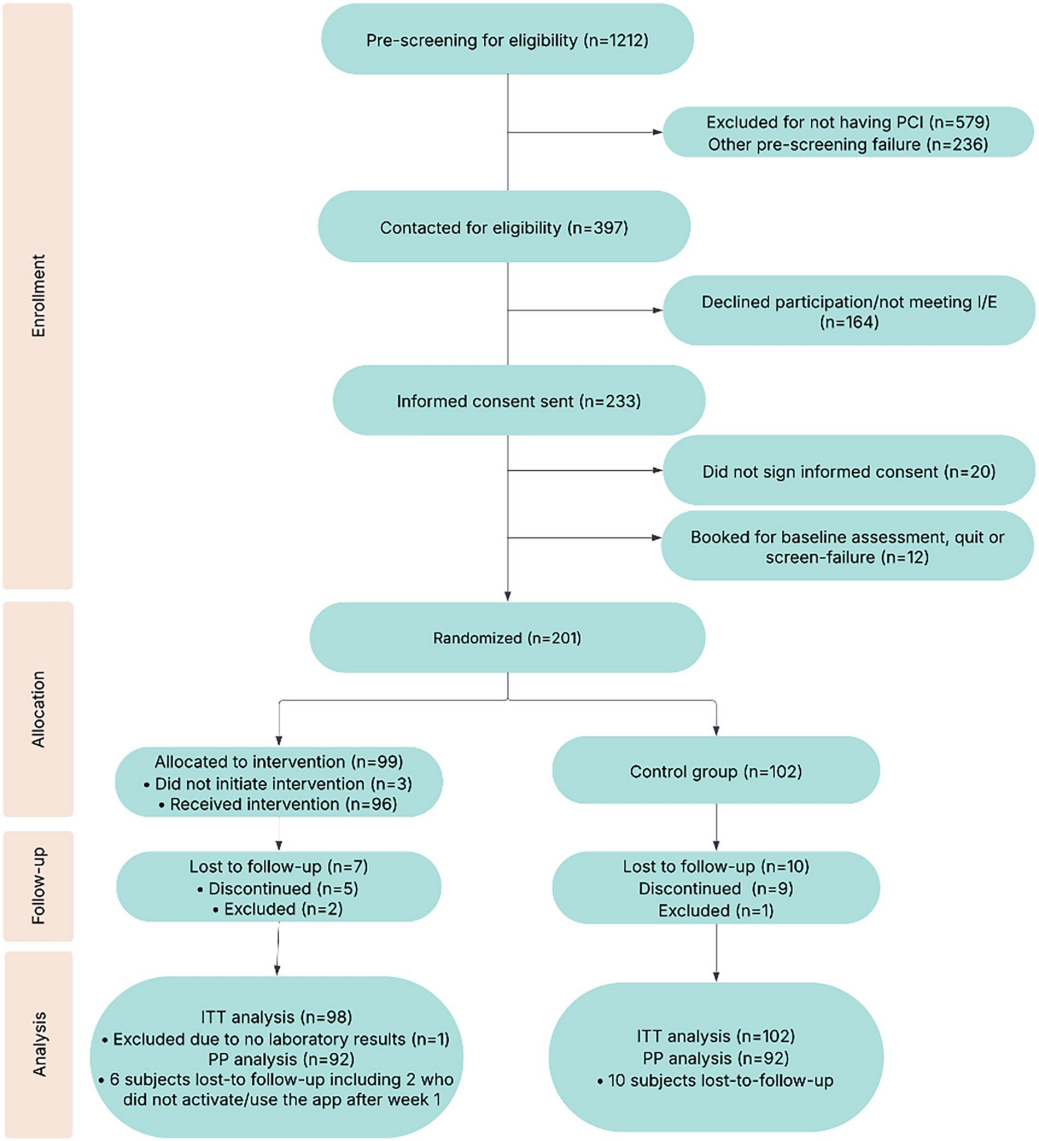
Study participant CONSORT flowchart. CONSORT, Consolidated Standards Of Reporting Trials; I/E, inclusion and exclusion criteria; ITT, intention-to-treat; n, number; PCI, percutaneous coronary intervention; PP, per-protocol.

Ultimately, 201 participants were successfully randomised. The first participant was enrolled on February 6th 2023. The ITT analysis included 200 participants (one individual did not complete baseline or follow-up), and the per-protocol (PP) analysis included 184 participants. Participant enrolment, allocation, and analysis flow are illustrated in Figure 2.

The mean age of the 200 participants was 64.4 years (SD 9.1), and 79.5% were male. Based on BMI, 42% were classified as overweight and 44% as obese. Nearly half of the participants were employed full-time (47.5%), and a majority had a history of hypertension (62%). For most participants (67.5%), the index PCI represented their first CAD diagnosis, and 65% reported a family history of CAD. Before PCI, 57% of participants had no troponin T elevation; 38.5% presented with stable angina or were ambulant, and 18.5% with unstable angina. Only 14 participants (7 in each group) were active smokers at baseline. Demographic and clinical characteristics at baseline are summarised in Table 1.

**Table 1:** Demographic and clinical characteristics at baseline.

Variable	All patients	Intervention	Control
	*n* = 200	*n* = 98	*n* = 102
Age in years, mean (SD)	64.4 (9.1)	63.9 (9.1)	64.8 (9.1)
Sex, *n* (%)			
Female	41 (20.5)	20 (20.4)	21 (20.6)
Male	159 (79.5)	78 (79.6)	81 (79.4)
Family status, *n* (%)			
Married	148 (74.0)	72 (73.5)	76 (74.5)
In cohabitation	18 (9.0)	9 (9.2)	9 (8.8)
Single	14 (7.0)	6 (6.1)	8 (7.8)
Divorced/separated	13 (6.5)	8 (8.2)	5 (4.9)
Widowed	7 (3.5)	3 (3.1)	4 (3.9)
Education status, *n* (%)			
Primary or less	42 (21.0)	19 (19.4)	23 (22.5)
Secondary/matriculate	12 (6.00)	7 (7.14)	5 (4.90)
Trades/vocational school	86 (43.0)	45 (45.9)	41 (40.2)
University degree	60 (30.0)	27 (27.6)	33 (32.4)
Work status, *n* (%)			
Not on the labour market	76 (38.0)	34 (34.7)	42 (41.2)
Part-time	29 (14.5)	14 (14.3)	15 (14.7)
Full-time	95 (47.5)	50 (51.0)	45 (44.1)
Smoking status, *n* (%)			
Current smoker	14 (7.00)	7 (7.14)	7 (6.86)
Former smoker	100 (50.0)	46 (47.0)	54 (53.0)
Never smoked	86 (43.0)	45 (45.9)	41 (40.2)
BMI categories, *n* (%)			
Healthy weight (BMI 18.5–24.9)	28 (14.0)	19 (19.4)	9 (8.82)
Overweight (BMI 25–29.9)	84 (42.0)	42 (42.9)	42 (41.2)
Obesity (BMI >30)	88 (44.0)	37 (37.8)	51 (50.0)
Blood pressure in mmHg, mean (SD)			
Systolic	135.7 (15.8)	135.0 (14.9)	136.4 (16.6)
Diastolic	79.3 (8.6)	79.7 (8.1)	79.0 (9.1)
Subject's disease status before enrolment, *n* (%)			
Stable angina or ambulant	77 (38.5)	39 (39.8)	38 (37.3)
Unstable angina	37 (18.5)	18 (18.4)	19 (18.6)
STEMI	36 (18.0)	15 (15.3)	21 (20.6)
NSTEMI	50 (25.0)	26 (26.5)	24 (23.5)
First diagnosis of CAD, *n* (%)	135 (67.5)	71 (72.4)	64 (62.7)
Family history of CAD, *n* (%)	130 (65.0)	68 (69.4)	62 (60.8)

Comorbidities, *n* (%)			
Hypertension	124 (62.0)	54 (55.1)	70 (68.6)
Hypercholesterolemia	82 (41.0)	40 (40.8)	42 (41.2)
Diabetes (type I or II)	35 (17.5)	19 (19.3)	16 (15.7)
Sleep apnoea	28 (14.0)	15 (15.3)	13 (12.7)
Anxiety disorder	24 (12.0)	11 (11.2)	13 (12.7)
Depression	20 (10.0)	8 (8.16)	12 (11.8)
Atrial fibrillation	19 (9.50)	10 (10.2)	9 (8.82)

BMI, body mass index; CAD, coronary artery disease; n, number; NSTEMI, non-ST-elevation myocardial infarction; SD, standard deviation; STEMI, ST-elevation myocardial infarction.

**Figure 3:**
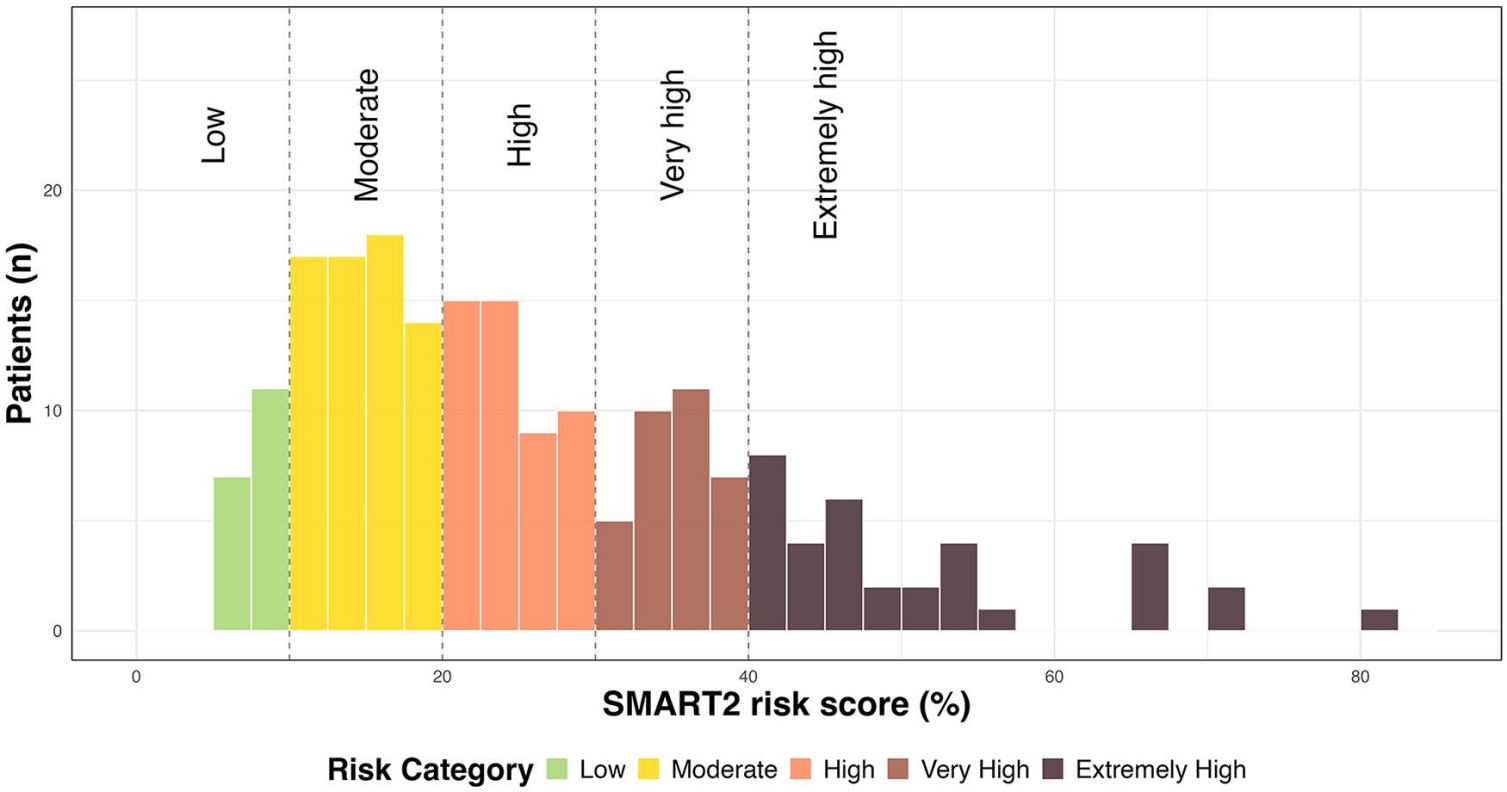
Distribution and risk categorisation of SMART2 scores at baseline. *n*, number; SMART2, Second Manifestations of ARTerial disease, version 2.

The overall median SMART2 score at baseline was 22.6% (IQR 14.6–35.6), with 33% of patients classified as moderate risk, 24.5% as high risk, 16.5% as very high risk, and 17% as extremely high risk. The baseline values of outcome measures are shown in Table 2. The distribution of SMART2 scores is presented in Figure 3.

**Table 2:** Baseline values of the outcomes by randomization group.

Variable	Intervention	Control
	*n* = 98	*n* = 102
Primary endpoint		
SMART2 risk score [0–100], median (IQR)	21.2 (14.2, 35.8)	24.4 (15.4, 35.4)
SMART2 risk categories, *n* (%)		
Low (<10)	12 (12.2)	6 (5.9)
Moderate (≥10 to <20)	32 (32.7)	34 (33.3)
High (≥20 to <30)	25 (25.5)	24 (23.5)
Very high (≥30 to <40)	10 (10.2)	23 (22.5)
Extremely high (≥40)	19 (19.4)	15 (14.7)
Secondary endpoints		
CADE-Q SV [0–20], mean (SD)	14.4 (3.4)	14.5 (3.2)
Clinical aspects [0–4]	2.7 (0.8)	2.6 (0.7)
Risk factors [0–4]	3.0 (0.8)	3.1 (0.8)
Exercise [0–4]	2.9 (1.0)	2.9 (1.0)
Diet [0–4]	3.3 (1.1)	3.2 (1.1)
Psychosocial risk [0–4]	2.6 (1.1)	2.6 (0.9)
MMAS-8 [0–8], mean (SD)	7.7 (0.7)	7.6 (0.7)
Low [0–5.9], *n* (%)	4 (4.1)	1 (1.0)
Moderate [6–7.9], *n* (%)	18 (18.4)	32 (31.4)
High [8], *n* (%)	76 (77.6)	69 (67.6)
DASS-21 [0–126], median (IQR)	13.0 (8.0, 24.0)	12.0 (6.0, 28.0)
Depression scale [0–42]	4.0 (2.0, 8.0)	4.0 (2.0, 10.0)
Anxiety scale [0–42]	4.0 (2.0, 6.0)	3.0 (0.0, 6.0)
Stress scale [0–42]	4.0 (2.0, 10.0)	6.0 (2.0, 12.0)
SAQ-7 [0–100], mean (SD)	64.5 (19.0)	62.4 (19.4)
Physical limitation scale [0–100]	62.6 (21.2)	61.4 (23.7)
Angina frequency scale [0–100]	78.0 (18.4)	75.1 (21.4)
Quality of life scale [0–100]	53.1 (27.3)	50.9 (27.1)
Exploratory endpoints		
EQ-5D-5L [0–100], mean (SD)	88.3 (13.8)	87.7 (13.7)
EQ-VAS [0–100], mean (SD)	76.5 (15.7)	71.0 (18.2)
Weight in kg, mean (SD)	90.4 (14.8)	93.5 (16.7)
BMI in kg/m^2^, mean (SD)	29.1 (4.3)	30.2 (4.3)
Waist circumference in cm, mean (SD)	106.3 (11.5)	108.7 (10.9)
Waist-height ratio, mean (SD)	0.6 (0.1)	0.6 (0.1)
Sit-to-stand test: repetitions, mean (SD)	12.7 (3.6)	13.4 (3.7)
(Missing)	0	2
Metabolic syndrome conditions [0–5], mean (SD)	2.6 (1.1)	2.6 (1.1)
Lipoprotein (a) in nmol/L, median (IQR)	21.5 (9.0, 145.0)	18.5 (7.0, 53.0)
High-sensitivity CRP in mg/L, median (IQR)	1.8 (0.8, 3.7)	1.2 (0.7, 2.8)
(Missing)	2	5
eGFR in mL/min/1.73 m², mean (SD)	70.8 (14.7)	70.7 (15.7)
HDL cholesterol in mmol/L, mean (SD)	1.3 (0.3)	1.3 (0.3)
Triglyceride/HDL ratio, median (IQR)	0.9 (0.6, 1.4)	0.9 (0.6, 1.4)
Liver function tests in U/L, median (IQR)		
ALT	26.0 (20.0, 39.0)	26.0 (21.0, 35.0)
AST	23.0 (19.0, 26.0)	23.5 (20.0, 28.0)
GGT	31.0 (22.0, 45.0)	28.5 (18.0, 47.0)
(Missing)	1	0

ALT, alanine transaminase; AST, aspartate aminotransferase; BMI, body mass index; CADE-Q SV, 20-item Coronary Artery Disease Education Questionnaire—Short Version; CRP, C-Reactive Protein; DASS-21, 21-item depression, anxiety and stress scale; eGFR, estimated Glomerular Filtration Rate; EQ-5D-5L, five-level EuroQol-5D questionnaire; EQ-VAS, EQ visual analogue scale; GGT, gamma-glutamyl transferase; HDL, high-density lipoprotein; IQR, interquartile range; MMAS-8, eight-item Morisky Medication Adherence Scale; n, number; SAQ-7, seven-item Seattle Angina Questionnaire; SD, standard deviation.

### User retention, engagement, and satisfaction with the SKH app

**Figure 4:**
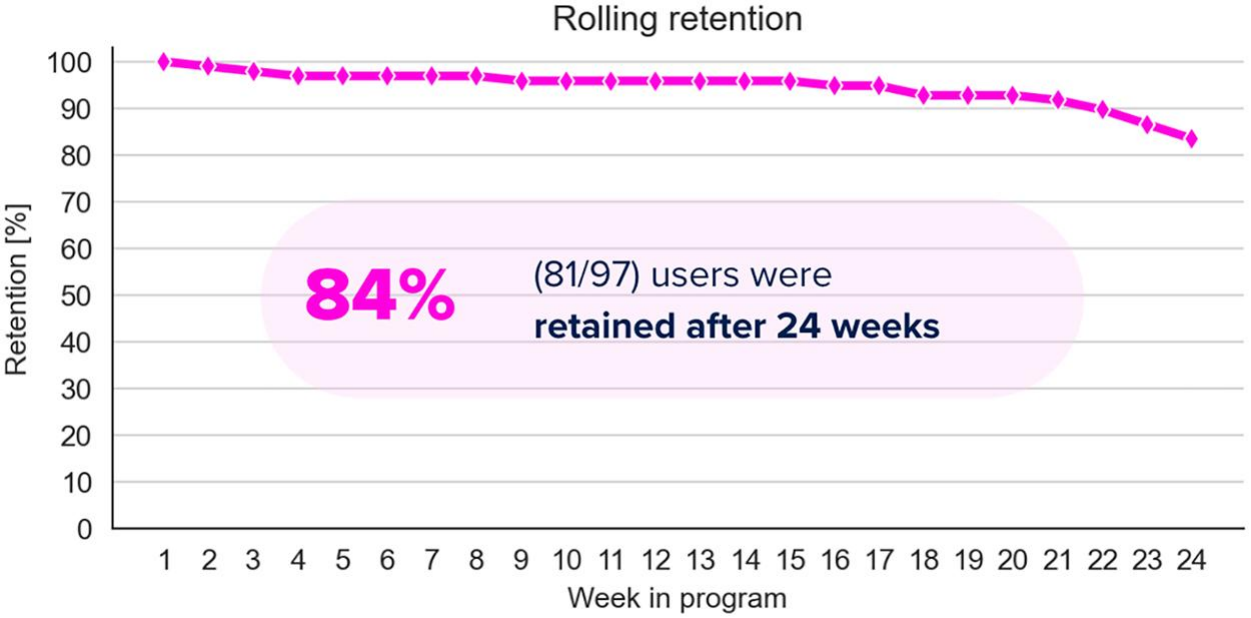
Rolling retention of the SKH app users over the main study period (6 months).

User retention and engagement with the SKH app were high during the 24-week program. A total of 83 out of 97 users (86%) who activated the app finished 75% of the program, and 69 users (71%) remained active for all 24 weeks. Median app usage was 138/168 days (IQR 88–166), equivalent to 5.75 active days per week (IQR 3.67–6.92). Over half of the users (57%) were highly engaged, defined as using the app at least five days per week for 18 weeks. Task compliance averaged 67%, and 81 users (84%) were retained at week 24 (Figure 4).

**Figure 5:**
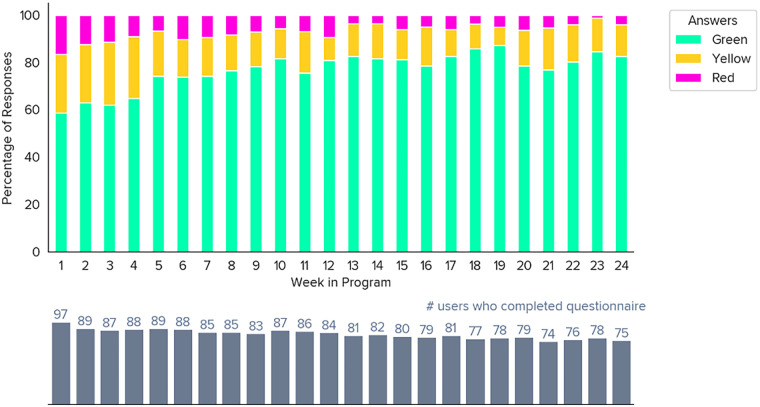
In-app survey results over 6 months from the RPM questionnaire.

Weekly RPM survey completion averaged 94%, with a significant reduction in high-severity symptom reports over time, where the proportion of users reporting low-severity symptoms increased from 57% at Week 1 to 83% at Week 24 (McNemar's test, *P* < 0.001) (Figure 5). Nearly half of the users (48%) set up medication reminders. At 6 months, satisfaction survey responses were highly positive: 93% found the app well organised, 88% found it easy to use, and 83% would recommend it. The most valued features were educational materials (85%), physical activity tracking (81%), and symptom tracking (71%).

### SMART2 risk score reduction

The primary endpoint was the baseline-adjusted between-group difference in the SMART2 risk score at 6 months, expressed as predicted ARR. At 6 months, the adjusted ARR was −0.3%, and at 12 months −0.9%; neither difference was significant (Table 3). Unadjusted models yielded slightly larger reductions, but these were not significant. Therefore, the primary endpoint was not met.

**Table 3:** Results of estimated changes from baseline and between-group differences in primary, secondary and exploratory endpoints for the ITT sample.

**Variable**	**Time point**	**Adjusted group mean (95% CI)** *		**Difference, intervention vs. control (95% CI)** *	***P*-value** **
		**Intervention**	**Control**		
Primary endpoint					
SMART2 risk score, % (unadjusted)^a^	Month 6	23.9 (22.3, 25.6)	24.4 (22.8, 26.1)	−0.5 (−2.8, 1.9)	0.680
	Month 12	23.2 (21.6, 24.9)	24.4 (22.8, 26.1)	−1.2 (−3.6, 1.2)	0.333
SMART2 risk score, % (adjusted)^a^	Month 6	23.9 (22.2, 25.8)	24.2 (22.4, 26.0)	−0.3 (−2.4, 1.9)	0.821
	Month 12	23.3 (21.5, 25.1)	24.2 (22.5, 26.0)	−0.9 (−3.2, 1.3)	0.409
Secondary endpoints					
CADE-Q SV [0–20]^b^	Month 6	16.6 (16.0, 17.1)	14.9 (14.4, 15.4)	1.6 (1.0, 2.2)	**<0**.**001**
	Month 12	16.4 (15.9, 16.9)	15.1 (14.5, 15.6)	1.4 (0.7, 2.0)	**<0**.**001**
MMAS-8 [0–8]^c^	Month 6	7.85 (7.74, 7.95)	7.70 (7.58, 7.82)	0.14 (0.01, 0.28)	**0**.**030**
	Month 12	7.81 (7.70, 7.92)	7.78 (7.66, 7.89)	0.04 (−0.10, 0.17)	0.604
DASS-21 [0–126]^c^	Month 6	14.4 (12.3, 16.6)	16.6 (14.4, 18.8)	−2.2 (−4.9, 0.6)	0.132
	Month 12	13.7 (11.6, 15.8)	14.9 (12.7, 17.0)	−1.2 (−4.0, 1.6)	0.403
SAQ-7 [0–100]^b^	Month 6	76.5 (73.4, 79.5)	78.1 (75.0, 81.1)	−1.6 (−5.4, 2.2)	0.408
	Month 12	78.4 (75.3, 81.6)	78.5 (75.5, 81.5)	−0.1 (−3.9, 3.7)	0.963
Exploratory endpoints					
EQ-5D-5L [0–100]^c^	Month 6	90.6 (88.9, 92.3)	89.4 (87.7, 91.1)	1.2 (−0.9, 3.2)	0.257
	Month 12	90.7 (89.0, 92.4)	89.9 (88.1, 91.6)	0.8 (−1.3, 2.9)	0.464
EQ-VAS [0–100]^c^	Month 6	79.9 (77.5, 82.4)	79.2 (76.7, 81.6)	0.7 (−2.4, 3.8)	0.644
	Month 12	80.5 (78.0, 82.9)	79.4 (76.9, 81.8)	1.1 (−2.0, 4.2)	0.476
Weight, in kg^c^	Month 6	90.4 (89.6, 91.2)	91.6 (90.8, 92.3)	-1.2 (−2.2, −0.2)	**0**.**015**
	Month 12	91.0 (90.1, 91.8)	91.6 (90.9, 92.4)	−0.6 (−1.6, 0.3)	0.202
BMI, in kg/m^2^^c^	Month 6	29.2 (29.0, 29.5)	29.6 (29.3, 29.8)	−0.3 (−0.7, 0.0)	**0**.**036**
	Month 12	29.4 (29.2, 29.7)	29.6 (29.3, 29.9)	−0.2 (−0.5, 0.2)	0.325
Waist-to-height ratio^c^	Month 6	0.60 (0.59, 0.60)	0.61 (0.60, 0.61)	−0.01 (−0.02, −0.00)	**0**.**007**
	Month 12	0.60 (0.59, 0.61)	0.61 (0.60, 0.61)	−0.01 (−0.01, 0.00)	0.106
Sit-to-stand test: repetitions^c^	Month 6	15.8 (15.0, 16.6)	15.1 (14.3, 15.9)	0.7 (−0.3, 1.7)	0.169
	Month 12	17.2 (16.4, 18.0)	16.6 (15.7, 17.4)	0.7 (−0.4, 1.7)	0.205
Metabolic syndrome conditions [0–5]^b^	Month 6	2.44 (2.22, 2.67)	2.43 (2.21, 2.64)	0.02 (−0.26, 0.30)	0.899
	Month 12	2.35 (2.13, 2.58)	2.40 (2.18, 2.62)	−0.04 (−0.32, 0.23)	0.757
Lipoprotein (a) in nmol/L^c^	Month 6	69.9 (68.0, 71.8)	69.9 (68.0, 71.9)	−0.1 (−2.5, 2.4)	0.970
	Month 12	68.9 (66.9, 70.9)	67.7 (65.7, 69.6)	1.2 (−1.3, 3.7)	0.340
High-sensitivity CRP in mg/L^c^	Month 6	1.29 (1.03, 1.56)	1.41 (1.16, 1.67)	−0.12 (−0.45, 0.22)	0.492
	Month 12	1.27 (1.00, 1.53)	1.45 (1.19, 1.71)	−0.18 (−0.51, 0.15)	0.277
eGFR in mL/min/1.73 m²^c^	Month 6	70.5 (69.2, 71.9)	69.9 (68.7, 71.2)	0.6 (−1.0, 2.2)	0.461
	Month 12	70.1 (68.8, 71.4)	69.4 (68.1, 70.7)	0.7 (−0.9, 2.3)	0.380
Systolic blood pressure in mmHg^b^	Month 6	133.8 (129.7, 138.0)	134.4 (130.2, 138.4)	−0.5 (−4.3, 3.3)	0.804
	Month 12	132.2 (128.0, 136.4)	134.0 (130.0, 138.0)	−1.8 (−5.5, 2.0)	0.357
Diastolic blood pressure in mmHg^b^	Month 6	76.3 (74.4, 78.2)	79.6 (77.7, 81.5)	−3.3 (−5.7, −0.9)	**0**.**007**
	Month 12	76.5 (74.5, 78.5)	80.2 (78.3, 82.2)	−3.7 (−6.1, −1.3)	**0**.**003**
HDL cholesterol in mmol/L^c^	Month 6	1.36 (1.32, 1.41)	1.39 (1.34, 1.43)	−0.02 (−0.08, 0.03)	0.391
	Month 12	1.38 (1.34, 1.43)	1.42 (1.38, 1.47)	−0.04 (−0.09, 0.02)	0.217
ALT in U/L^c^	Month 6	27.7 (25.9, 29.5)	27.7 (25.8, 29.6)	0.0 (−2.3, 2.3)	0.997
	Month 12	27.4 (25.5, 29.2)	26.8 (25.0, 28.6)	0.6 (−1.7, 2.8)	0.622
AST in U/L^c^	Month 6	23.5 (22.4, 24.6)	24.3 (23.2, 25.4)	−0.8 (−2.2, 0.6)	0.285
	Month 12	23.6 (22.5, 24.7)	23.9 (22.9, 25.0)	−0.3 (−1.7, 1.1)	0.657
GGT in U/L^c^	Month 6	38.4 (35.8, 41.0)	40.5 (37.8, 43.2)	−2.1 (−5.2, 1.0)	0.181
	Month 12	38.2 (35.7, 40.7)	39.2 (36.3, 42.2)	−1.1 (−4.2, 2.1)	0.517

* Endpoints were adjusted for baseline to correct for pre-existing baseline differences and additionally for covariates: age, sex, reason for PCI before enrolment, and indication if the enrolment PCI is the subject's first diagnosis of CAD.

** *P*-values below 0.05 are presented in bold.

a Analysed using a generalised linear mixed-effects model with a beta distribution and logit link.

b Analysed using linear mixed-effects regression model.

c Analysed using robust linear mixed-effects regression model.

ALT, alanine transaminase; AST, aspartate aminotransferase; BMI, body mass index; CAD, coronary artery disease; CADE-Q SV, 20-item Coronary Artery Disease Education Questionnaire—Short Version; CI, confidence interval; DASS-21, 21-item depression, anxiety and stress scale; CRP, C-Reactive Protein; eGFR, estimated glomerular filtration rate; EQ-5D-5L, five-level EuroQol-5D questionnaire; EQ-VAS, EQ visual analogue scale; GGT, gamma-glutamyl transferase; HDL, high-density lipoprotein; MMAS-8, eight-item Morisky Medication Adherence Scale; PCI, percutaneous coronary intervention; SAQ-7, seven-item Seattle Angina Questionnaire.

*Post hoc* subgroup analysis examined between-group differences in SMART2 risk scores stratified by BMI class. Among overweight participants (*n* = 84), a significant difference was observed at 12 months, with the intervention group showing a 3.7% greater absolute reduction in SMART2 risk score compared to the control group (mean difference −3.7%; 95% CI: −7.0% to −0.3%; *P* = 0.033) (Supplementary Table S1).

### Changes in patient-reported outcomes

The secondary endpoints included several patient-reported outcomes, such as disease knowledge, medication adherence, anxiety levels, depression, and stress, and disease burden in patients with CAD.

Significant between-group differences were observed in disease knowledge, as assessed by the CADE-Q SV (scale 0–20), with SKH app users showing greater improvements at 6 months (mean difference: 1.6; 95% CI: 1.0–2.2; *P* < 0.001) and 12 months (1.4; 95% CI: 0.7–2.0; *P* < 0.001) compared to the control group (Table 3). Improvements were also evident across most CADE-Q SV subdomains, particularly in psychosocial risk, diet, exercise, and risk factors, with significant between-group differences observed at both time points. No significant changes were found in the medical condition subdomain. Supplementary Figure S1 illustrates estimated within-group changes in the overall CADE-Q SV score and its five subdomains over time, based on model-derived means. Cronbach's alpha for the CADE-Q SV ranged from 0.73 to 0.76 across time points.

A *post hoc* subgroup analysis of CADE-Q SV scores among intervention participants showed that those who were highly engaged at 6 months (*n* = 55; used the app at least five days per week for 18 weeks) achieved significantly greater improvements in disease knowledge compared to less engaged participants (*n* = 42), with a mean difference of 0.90 points (95% CI: 0.06–1.73; *P* = 0.035) at 6 months and 1.27 points (95% CI: 0.43–2.11; *P* = 0.003) at 12 months (results not shown). Missing data were not imputed for this subgroup analysis.

A significant between-group difference was observed at 6 months for medication adherence (mean difference: 0.14; 95% CI: 0.01–0.28; *P* = 0.030), reflecting improved adherence among participants in the intervention group. No significant difference was found at 12 months (*P* = 0.604) (Table 3). Additionally, participants in the intervention group who reported maximum medication adherence at baseline showed a significantly smaller decline at 6 months compared to the control group, with a mean difference of 0.07 points (95% CI: 0.01–0.12; *P* = 0.015; data not shown). Cronbach's alpha for the MMAS-8 ranged from 0.253 to 0.446 across time points. Transitions between adherence categories are shown in Supplementary Figure S3.

No significant between-group differences were observed at either time point in anxiety, depression, or stress based on the overall scores or subdomains of the DASS-21, and in angina-related quality of life based on the scores of the SAQ-7 (Table 3).

For hospital admissions and emergency room (ER) visits, the summary counts (Table 4) and unadjusted survival probabilities were similar between the intervention and control groups (log-rank *P* = 0.62 and *P* = 0.52, respectively). For hospital admissions, the adjusted Cox proportional hazards model showed no significant effect from the intervention [hazard ratio (HR) = 0.89, 95% CI: 0.32–2.43, *P* = 0.815]. Similarly, for ER visits, no significant differences were found between groups after adjustment (HR = 0.86, 95% CI: 0.50–1.48, *P* = 0.587).

**Table 4:** Hospital utilisation metrics, summed per group, in the periods 12 months prior to baseline and 12 months after baseline.

**Metric**	**Time period**	**Group**	**Total visits or admissions length**	**Unique Subjects with event**	**Avg. per subject with event** ^a^	**Avg. per group** ^b^
Number of admissions	Before (12 m)	Control	54	51	1.1	0.53
		Intervention	51	48	1.1	0.52
	After (12)	Control	9	8	1.1	0.09
		Intervention	10	10	1	0.1
Admission days	Before (12 m)	Control	143	51	2.8	1.4
		Intervention	112	48	2.3	1.14
	After (12 m)	Control	22	8	2.8	0.22
		Intervention	24	10	2.4	0.24
ER Visits	Before (12 m)	Control	65	47	1.4	0.64
		Intervention	69	50	1.4	0.7
	After (12 m)	Control	41	29	1.4	0.4
		Intervention	45	30	1.5	0.46
Outpatient Clinic	Before (12 m)	Control	30	22	1.4	0.29
		Intervention	24	23	1	0.24
	After (12 m)	Control	78	40	2	0.76
		Intervention	105	52	2	1.07

a Represents the average only among the subjects who had an event (e.g., respective total number of visits or admission days/unique subjects with event).

b Represents the population average across the entire randomised group (e.g., Total Admission Days/total group). Control group *n* = 102; Intervention group *n* = 98.

Avg, average; ER, emergency room; m, months.

In contrast, for outpatient clinic visits, the observed counts (105 intervention vs. 78 control) and unadjusted data suggested significantly more visits for the intervention group (log-rank *P* = 0.047). However, after adjusting for prior visit frequency, the intervention effect was not significant (HR = 1.53, 95% CI: 0.99–2.37, *P* = 0.054). Notably, across all three utilisation categories, cumulative pre-baseline events remained a highly significant predictor of post-baseline utilisation (*P* < 0.001).

### Exploratory endpoints

Exploratory outcomes showed no significant between-group differences in health-related quality of life as measured by EQ-5D-5L index scores or EQ-VAS at either 6 or 12 months. Small but significant differences were observed in body weight (−1.2 kg; *P* = 0.015), BMI (−0.3 kg/m^2^; *P* = 0.036), and waist-to-height ratio (−0.009; *P* = 0.007) at 6 months, favouring the intervention group, although these differences were not sustained at 12 months. Diastolic blood pressure was significantly lower in the intervention group at both time points (−3.3 mmHg at 6 months, *P* = 0.007; −3.7 mmHg at 12 months, *P* = 0.003). No significant differences were found for other exploratory measures, including sit-to-stand performance, number of metabolic syndrome conditions, lipoprotein(a), hs-CRP, eGFR, systolic blood pressure, HDL cholesterol, or liver enzymes (ALT, AST, GGT) at either time point (Table 3).

A *post hoc* analysis of eGFR, in which participants were categorised into five chronic kidney disease (CKD) stages (28), revealed that those in CKD stages 3 and 4 within the intervention group improved more than controls. At 6 months, the between-group difference was 2.9 mL/min/1.73 m^2^ (95% CI: −0.28 to 6.07; *P* = 0.074), and at 12 months the difference was significant at 3.9 mL/min/1.73 m^2^ (95% CI: 0.61–7.13; *P* = 0.020). Models were only adjusted for baseline eGFR. Baseline CKD stage distribution and longitudinal changes in eGFR among participants with CKD stages 3 and 4, stratified by randomisation group, are shown in Supplementary Figure S2.

### Per-protocol analysis

The PP analysis results were largely consistent with the ITT results, with neither analysis demonstrating a significant difference for the primary endpoint. The PP analysis mirrored the ITT analysis results across secondary outcomes, confirming the robustness of the study's observations regarding behaviour and knowledge. For details, see the **Per-protocol analysis** in the Supplementary text.

### Safety

Approximately 57% of patients overall experienced at least one adverse event (AE). Most AEs were mild or moderate in both groups, with severe AEs accounting for 15.7% of events in the intervention group and 8.6% in the control group (Supplementary Table S2).

The most common AEs were cardiac disorders (44% of all events), primarily consisting of chest pain investigations (45% of cardiac events), coronary interventions/stents (32%), and arrhythmias (15%). Other notable AE categories included infections (7.4%), nervous system disorders (5.1%), and metabolic disorders (5.1%). Serious adverse events (SAEs) were observed in 17.3% of patients in the intervention group and 14.7% in the control group. No AEs were attributed to the digital intervention.

## Discussion

The digital intervention in this RCT was very well received by participants, as evidenced by high compliance in the lifestyle support modules and high retention rates in the RPM. Although the SMART2 risk score did not improve at 6 months, an exploratory analysis revealed an overall positive effect in overweight participants receiving the digital intervention. Furthermore, in the overall intervention group, improvements were observed in several secondary endpoints such as symptoms, disease knowledge, and medication adherence when compared to SoC alone.

Low retention rates are a major challenge for lifestyle modification programs. In our study, we found a retention rate of 84%, which is high compared to other studies as a meta-analysis of 17 trials with apps for managing chronic diseases found a retention rate of 57% (29). Our high retention may in part be driven by the program's multimodal design, which incorporates evidence-based behavioural change techniques, gamification, healthcare professional communication, and RPM. As highlighted in recent literature, integrating interactive features and personalized support mechanisms provides a critical framework for sustaining long-term preventive behaviours (30).

Low participant engagement is another challenge for lifestyle modification programs. An extensive study with over a million users of a weight-loss app found that, on average, users remained engaged for only 29 days (31). We found that, from the start of the program, “Completers”, defined as those who completed 75% of the program or 18/24 weeks, were also more engaged than the “Drop-outs”, defined as those not returning to the app in any subsequent weeks after stopping. This may emphasise the importance of motivating users early in programs to maximise retention.

The lack of significant between-group differences in SMART2 risk scores may perhaps be attributed to the high-quality guideline-recommended SoC that all patients who had recently undergone PCI for their CAD received at the cardiology outpatient clinic. Prior modelling of optimal treatment outcomes demonstrates that even if all modifiable risk factors were addressed according to guideline targets, a substantial proportion of patients would still be classified at higher risk. Specifically, approximately one-fifth of patients would remain at a 10-year risk exceeding 20%, underscoring that residual risk remains an unmet challenge in secondary prevention (27). In contemporary cardiovascular care, the benefit of digital interventions on top of optimised interventional and pharmacological therapy might be difficult to detect within just a one-year timeframe. Future research should consider longer follow-up periods and real-world evidence or focus on populations with higher baseline risk to better capture the long-term preventive impact of digital lifestyle modifications. Our exploratory subgroup analysis of changes in the SMART2 risk score by BMI categories revealed that among overweight participants, the intervention group showed a significant reduction in SMART2 risk score compared to the control group. It is plausible that overweight participants found the lifestyle advice, especially relating to exercise, easier to adopt than those living with obesity.

The observed increase in disease knowledge in the intervention group suggests that although the SMART2 risk score did not improve, the program successfully empowered patients with the necessary knowledge for long-term self-management. Improvements were specifically noted in the domains of psychosocial risk, diet, exercise, and risk factors. This is critical because a gap often exists between clinical guidelines and the implementation of lifestyle changes. Improved knowledge in these areas addresses the modifiable risk factors that contribute to 90% of myocardial infarction risk (2). Furthermore, 57% of users were classified as “highly engaged,” which correlated with better outcomes in disease knowledge. These findings are supported by a recent 36-month evaluation of a home-based cardiac telerehabilitation program (32). Similar to our observation that “highly engaged” users achieved greater improvements in disease knowledge, the telerehabilitation program showed a dose-response relationship where lower adherence resulted in significantly lower disease knowledge. Furthermore, their long-term follow-up revealed that sustained improvements in disease awareness were associated with reduced long-term major adverse cardiac events (MACEs) (32). Therefore, sustained acquisition of disease knowledge may be a critical foundational step for long-term behavioural maintenance and eventual reduction of hard clinical events, even if these benefits were not yet fully captured by the 12-month SMART2 risk score.

The intervention did not improve the quality of life or psychological distress. However, the program significantly improved medication adherence at 6 months, driven by targeted education and digital reminders used by 48% of participants. This is in line with a systematic review of digital health solutions in CVD prevention, which found that reminders and personalised feedback promoted more consistent medication use (30). However, the loss of significance at 12 months suggests that sustained adherence may require prolonged behavioural reinforcement.

The RPM showed high clinical utility as indicated by high retention, engagement and reduction in high-severity symptoms (chest pain and shortness of breath). These findings suggest that the combination of digital education and regular symptom monitoring may help patients better manage their condition, improve symptom awareness, and recognise improvement early in their recovery process.

The intervention did not significantly reduce hospital admissions, unlike the significant reductions in readmission and MACEs in the HeartMed cohort (33). This likely stems from our smaller sample size and relatively short follow-up period, which is in keeping with conclusions from a systematic review on mobile health applications for secondary prevention after myocardial infarction or PCI (34). That review found that app-based interventions were associated with a significant reduction in unplanned hospital readmission compared to SoC and that greater benefits were observed with longer follow-up and higher adherence.

The hypothesis-generating exploratory finding of a sustained reduction in diastolic blood pressure suggests that long-term physiological benefits can be achieved. Blood pressure is one of the most frequently evaluated outcomes in digital CVD prevention, but results are often mixed, with some showing improvement and others showing parity with standard care (30). Our findings are echoed by the i-CARE study, in which a remotely delivered digital intervention for CAD patients also resulted in significant diastolic blood pressure reductions alongside improved self-care behaviours (35). Further *post hoc* hypothesis-generating exploratory subgroup findings were that renal function in participants in CKD stages 3 and 4 improved, suggesting that holistic support for diet, hydration, and medication adherence may specifically benefit kidney health in CAD patients. These findings should be interpreted with caution and require validation in future trials.

This is our second RCT on the use of a digital intervention in patients with heart disease, having previously reported on a study of outpatients with heart failure (36), and it is useful to consider what lessons were learned. A key lesson from both trials is how extremely well the digital intervention was received, not least the RPM. Also, in both trials, the average age of patients was around 65 years, with several participants in their seventies. This suggests that age is not a large obstacle for this new approach, which bodes well for the potential introduction of digital tools into clinical practice.

Digital tools are of particular importance as a global shortage of healthcare workers is expected, and a clear need is emerging for managing the prioritisation of this limited resource (37). A recent narrative review found that digital solutions generally achieved clinical and behavioural outcomes comparable to those of centre-based cardiac rehabiliation (38). The adoption of digital solutions can transform healthcare services, partly by replacing outpatient clinic visits with remote monitoring of individuals with chronic diseases but a stable clinical course. Digital solutions could lead to more frequent monitoring and follow-up than spreading outpatients' visits to every 6–12 months. RPM might also have the added benefit of detecting a clinical exacerbation at an earlier stage if the monitoring is relatively frequent.

In our two trials there were also suggestions of clinical benefits, although both included mostly stable well-treated patients, and a follow-up time only one year. In the heart failure trial, improved self-care and disease knowledge, along with positive changes in key metabolic parameters were observed, while in the CAD trial, lowered blood pressure and improved symptoms were observed. In both trials, we were able to identify subgroups in which *post hoc* benefits in the clinical endpoints were significantly improved.

Digital solutions utilising gamified approaches and behavioural sciences are a good way to promote a healthy lifestyle, as the patients can focus on this on their own time and in their environment. This also provides scalable ways of providing support, motivation, and feedback.

### Strengths and limitations

A strength of the study was that the digital program was designed by a team of behavioural and medical experts based on the latest evidence-based clinical guidelines (5, 6), behaviour change theories, and gamification (39). This ensured that the set goals measured outcomes relevant for CAD patients, while the app itself was easy to use and engaging. This is in line with a review of mobile health apps for secondary prevention in CVD risk patients, which found that gamified apps resulted in more improvement than comparators or a neutral control app (40).

Study limitations included a relatively short follow-up duration, insufficient to evaluate the intervention's impact on hard clinical endpoints, and a study population already optimised on high-intensity SoC, as all patients had just undergone a PCI and were receiving guideline-recommended medication. A notable methodological limitation of this study is that blinding of patients, nurses, and physicians was not feasible due to the interactive nature of the digital intervention. In traditional clinical trials, a double-blind process is the gold standard to minimize bias where patients might alter their behaviour or self-reported outcomes simply because they are aware they are receiving a novel intervention. However, masking participant engagement with a smartphone application and remote health coaching remains a challenge in digital therapeutics. To mitigate potential bias, the statistical analyses for this trial were performed by a statistician who remained strictly blinded to group allocation until the statistical report was finalized. Because no formal correction for multiple comparisons was applied, the exploratory results should be considered hypothesis-generating and interpreted with a degree of caution. Future research utilising larger cohorts, longer observation periods, and more targeted patient populations is warranted to fully establish the clinical efficacy of digital therapeutics in secondary CAD prevention.

Finally, excluding participants unable to operate a smartphone can introduce a selection bias, potentially omitting high-risk patients with lower digital literacy. This “digital divide” remains a significant obstacle in digital health (30). A critical area for future research is qualitative usability testing to identify the specific barriers these individuals face.

## Conclusions

This study suggests that patients who have recently undergone PCI for CAD have a high degree of acceptance of this novel digital health program. Future research needs to identify which specific subgroups may derive the most benefit from digital lifestyle interventions adjunct to SoC. Our exploratory results suggest that patients who are overweight or have moderate renal impairment are particularly responsive to the program. These findings could help clinicians target digital therapeutics to patients with specific metabolic and clinical profiles to maximise their impact in cardiovascular secondary prevention.

## Data Availability

The datasets presented in this article are not readily available because of restrictions in the informed consent form. Additional summary statistics will be provided upon reasonable request. Requests to access the datasets should be directed to davidar@landspitali.is.
