# Supplementary material for: Use of a digital health solution after percutaneous coronary intervention: a randomised controlled trial
Source: Front Digit Health. 2026 Jul 21;8:1851777. doi: 10.3389/fdgth.2026.1851777 (PMC13433347; doi:10.3389/fdgth.2026.1851777)
Supplement: Supplementary file 1 [file Datasheet1.docx]

# **Supplementary materials**

## **Per-protocol analysis**

The per-protocol (PP) analyses included 92 participants in both the intervention and control groups (**Figure 2 in manuscript**), allowing comparison with the ITT results. No significant differences were observed in the primary endpoint. At 6 months, the predicted ARR was 0.16% higher in the control group (*P* = 0.893), whereas at 12 months it was 5.32% greater in the intervention group (*P* = 0.652). Regarding secondary outcomes, the intervention group, similarly to ITT, showed a significant improvement in CADE-Q SV scores, with increases of 1.4 points at 6 months and 1.3 points at 12 months (both *P* < 0.001). Medication adherence was also higher in the intervention group by 0.15 points at 6 months (*P* = 0.027), but no difference was observed at 12 months (*P* = 0.704). The DASS-21 score decreased by 2.2 points more in the intervention group at 6 months, although this difference was not significant (*P* = 0.084). No significant differences were observed in SAQ-7 scores at either 6 months (*P* = 0.463) or 12 months (*P* = 0.981).

## **Supplementary Table S1 SMART2 risk scores stratified by BMI class**

| **Subgroup** | **Time point** | **Adjusted group mean (95% CI) in SMART2 score^*^** | | **Difference, intervention vs. control (95% CI)^*^** | ***P*-value^**^** |
| --- | --- | --- | --- | --- | --- |
|  |  | **Intervention** | **Control** |  |  |
| **Healthy weight**  (n = 28) | Month 6 | 24.5 (21.0, 28.4) | 22.9 (18.3, 28.2) | 1.6 (-4.5, 7.6) | 0.606 |
|  | Month 12 | 23.7 (20.2, 27.6) | 23.7 (19.1, 29.1) | 0.0 (-6.1, 6.1) | 0.992 |
| **Overweight**  (n = 84) | Month 6 | 24.1 (21.6, 26.7) | 25.3 (22.7, 28.1) | -1.2 (-4.7, 2.2) | 0.484 |
|  | Month 12 | 21.4 (19.1, 24.0) | 25.1 (22.5, 27.9) | -3.7 (-7.0, -0.3) | **0.033** |
| **Obesity**  (n = 88) | Month 6 | 23.6 (21.0, 26.5) | 23.5 (21.3, 25.9) | 0.1 (-3.2, 3.5) | 0.944 |
|  | Month 12 | 25.2 (22.4, 28.1) | 23.4 (21.1, 25.8) | 1.8 (-1.7, 5.2) | 0.318 |

*Endpoints were analysed with a beta mixed-effects regression model and adjusted for baseline to correct for pre-existing baseline differences and additionally for covariates: age, sex, reason for PCI before enrolment, and indication if the enrolment PCI is the subject’s first diagnosis of CAD.

** p-values below 0.05 are presented in bold.

CAD, coronary artery disease; CI, confidence interval; n, number; PCI, percutaneous coronary intervention.

## **Supplementary Table S2 Adverse events**

| **Variable** | **Intervention**  **n = 98** | **Control**  **n = 102** |
| --- | --- | --- |
| **Patients with any AE, n (%)** | 54 (55.1) | 60 (58.8) |
| **Patients with SAE, n (%)** | 17 (17.3) | 15 (14.7) |
| **Total number of any AEs, n** | 83 | 93 |
| **AE severity** |  |  |
| Mild, n (%) | 39 (47) | 40 (43) |
| Moderate, n (%) | 31 (37.3) | 45 (48.4) |
| Severe, n (%) | 13 (15.7) | 8 (8.6) |
| **Treatment-related AE, n** | 0 | 0 |
| **Total number of SAEs, n** | 21 | 17 |
| **SAE severity** |  |  |
| Mild, n (%) | 2 (9.5) | 3 (17.6) |
| Moderate, n (%) | 8 (38.1) | 10 (58.8) |
| Severe, n (%) | 11 (52.4) | 4 (23.5) |
| **Treatment-related SAE, n** | 0 | 0 |

AE, adverse event; SAE, serious adverse event; n, number.

## **Supplementary Figure S1 Changes in disease knowledge scores on the CADE-Q SV from baseline: summary and sub-score domains**

CADE-Q SV, short version of the Coronary Artery Disease Education Questionnaire; CI, confidence interval.

## **Supplementary Figure S2 Baseline CKD stage distribution and longitudinal changes in eGFR among participants with CKD stages 3 & 4 by randomisation group**

The outcome was analysed using a robust linear mixed-effects regression model and adjusted for baseline outcome only.

CI, confidence interval; CKD, chronic kidney disease; eGFR, estimated Glomerular Filtration Rate.

## **Supplementary Figure S3 Sankey flow of medication adherence trajectories (MMAS-8 categories)**

High adherence corresponds to a score of 8 on the MMAS-8; medium adherence ranges from 6 to 7.9; low adherence ranges from 0 to 5.9.

MMAS-8, 8-item Morisky Medication Adherence Scale; NA, not applicable.
